# Multi-sectoral prioritization of zoonotic diseases: One health perspective from Ahmedabad, India

**DOI:** 10.1371/journal.pone.0220152

**Published:** 2019-07-30

**Authors:** Sandul Yasobant, Deepak Saxena, Walter Bruchhausen, Farjana Zakir Memon, Timo Falkenberg

**Affiliations:** 1 Center for Development Research (ZEF), University of Bonn, Bonn, Germany; 2 Faculty of Medicine, University of Bonn, Bonn, Germany; 3 Indian Institute of Public Health Gandhinagar (IIPHG), Gujarat, India; 4 Institute of History and Ethics of Medicine, University of Cologne, Faculty of Medicine and University Hospital Cologne, Cologne, Germany; 5 GeoHealth Centre, Institute for Hygiene and Public Health, University Bonn, Bonn, Germany; Faculty of Science, Ain Shams University (ASU), EGYPT

## Abstract

**Background:**

Prioritizing zoonotic diseases is one of the emerging tasks for developing multi-sectoral collaboration within One Health. Globally, many efforts have been made to prioritize zoonotic diseases at national levels, especially in low resource settings. Prioritization of zoonoses has been conducted in different countries at different levels (i.e. national, regional and local) for different purposes. India has also initiated prioritization of zoonotic diseases at the national level. However, in a country like India with wide climatic variations, different animal-human and vector densities, it is important to look at these zoonotic conditions in local settings too. The present study aims to determine which zoonoses should be prioritized for collaboration between stakeholders in the Indian city of Ahmedabad.

**Methods:**

The present study followed a participatory research method, entailing a stakeholder workshop for prioritizing zoonotic diseases in Ahmedabad. It was carried out through a facilitated consultative process involving 19 experts in zoonoses from the human and animal health systems during a one-day workshop in September 2018. To prioritize the zoonotic diseases, the One Health Zoonotic Disease Prioritization (OHZDP) tool of the U.S. Centers for Disease Control and Prevention was adopted. The Analytical Hierarchical Process (AHP) and decision-tree analysis were used to rank the diseases.

**Results:**

Out of 38 listed zoonotic diseases, 14 were selected for prioritization. These were scored and weighed against five criteria: severity of disease in humans, potential for epidemic and/or pandemic, availability of prevention and/or control strategies, burden of animal disease existing inter-sectoral collaboration.

The top five diseases that have been prioritized for Ahmedabad are Rabies, Brucellosis, Avian Influenza (H5N1), Influenza A (H1N1) and Crimean-Congo Hemorrhagic Fever. Sensitivity analysis did not indicate significant changes in zoonotic disease prioritization based on criteria weights.

**Conclusion:**

Prioritization of zoonotic diseases at the local level is essential for development of effective One Health strategies. This type of participatory disease prioritization workshop is highly recommended and can be replicated in other Indian cities, as well as in other low and middle-income countries.

## Background

Emerging and re-emerging zoonotic diseases are increasing globally, particularly in places with high host species richness and a high intensity of contact between animals and humans, as well as those located in lower latitudes [1–3]. Multi-sectoral collaboration through a One Health approach is being popularized either for management or for effective prevention of zoonotic diseases [4–8]. However, there is no blueprint for implementing One Health in a specific setting because of extensive challenges in bringing multiple stakeholders of the human, animal and environmental health sectors together. The major challenge in multi-sectoral collaboration often is the unspecified roles and responsibilities of stakeholders, and poor governance [9,10]. Despite challenges, some initiatives have been taken at national [11] and local levels [12]. However, evidence suggests that such collaborations are limited to outbreaks and are not sustained in endemic periods [13]. To establish a sustained, proactive and routine system, prioritization of zoonotic conditions through multi-sectoral collaboration within the respective settings is of utmost importance. Joint prioritization of zoonoses should benefit for the efficient and effective surveillance, developing laboratory capacity, targeting efficient outbreak prediction, implementing common disease control strategies, and identifying integrated research activities across sectors: human, animal, environmental [14].

Historically, infectious disease prioritization was within the purview of public health officials [15,16]. However, with progress of public health strategies, prioritization became an important tool for various stakeholders to receive common funding or for implementing joint research projects. The approaches used to prioritize diseases are: qualitative, semi-quantitative or quantitative [17–19].

With respect to zoonoses, prioritization has been conducted at different sites, such as Congo [20], Ethiopia [21], Kenya [22], Tanzania [23], Uganda [24] and North America [25]. Similarly, in India there have been some efforts for prioritizing zoonotic diseases at the national level [26,27]. However, to date, there is no zoonoses prioritization documented at local levels, such as cities. It is important to prioritize these emerging zoonotic diseases, especially in rapidly growing cities. As part of the larger project: ‘Research in exploring Inter-sectoral Collaboration for One Health Approach’ (RICOHA), we conducted zoonotic disease prioritization in an Indian city, Ahmedabad.

Prioritizing zoonotic diseases at a local level will not only emphasize the most important diseases to focus on but can also facilitate the development of One Health collaboration between local stakeholders [14]. Ahmedabad has documented various zoonotic diseases, ranging from outbreaks of Crimean-Congo hemorrhagic fever [28] and bird flu [29], to the long epidemic of chikungunya [30] and the recent epidemic of Zika [31]. The present study aims to determine which zoonoses need to be prioritized for collaboration between stakeholders in Ahmedabad, India.

## Methods

This study is part of the comprehensive RICOHA study, which aims at developing a One Health convergence model in Ahmedabad. The detailed study methodology is described elsewhere [32]. The present paper adopts the already established participatory method (stakeholder workshop) for prioritizing zoonotic diseases in Ahmedabad, India. The methodology follows the instrument of the U.S. Centers for Disease Control and Prevention (CDC), i.e. One Health Zoonotic Disease Prioritization (OHZDP) tool [14], which was adopted to the local context. In 2014, the CDC developed the OHDZP tool to be used in situations where comprehensive quantitative data is not available [33]. Further information on the OHZDP tool can be found with the CDC [14].

Data was collected through a facilitated consultative process involving 19 experts in zoonoses from the human and animal health systems during a one-day workshop in September 2018. To select participants, institutions (government, research and academia) and departments that work on zoonoses in the areas of surveillance, research and diagnostics in either the human or animal health sector were identified (S1 Table). These were invited to nominate the most appropriate individual to attend the workshop. The process of contacting stakeholders was initiated three months prior to the workshop. Among the participants were: medical officer of health, epidemic officer, malaria officer, entomologist, microbiologists, surveillance officer from the human health system, zoo veterinarian, superintendent of cattle nuisance control department, foot and mouth disease laboratory director, animal husbandry department director, veterinarian responsible for zoonotic diseases from the human health surveillance system.

The process of OHZDP tool consists of five steps:

1. Identification of zoonoses to be prioritized
2. Development of five criteria to prioritize diseases
3. Development of questions with categorical answers for each criterion based on available data
4. Weighting of the criteria
5. Ranking of the zoonoses using a decision tree analysis

**Fig 1:**
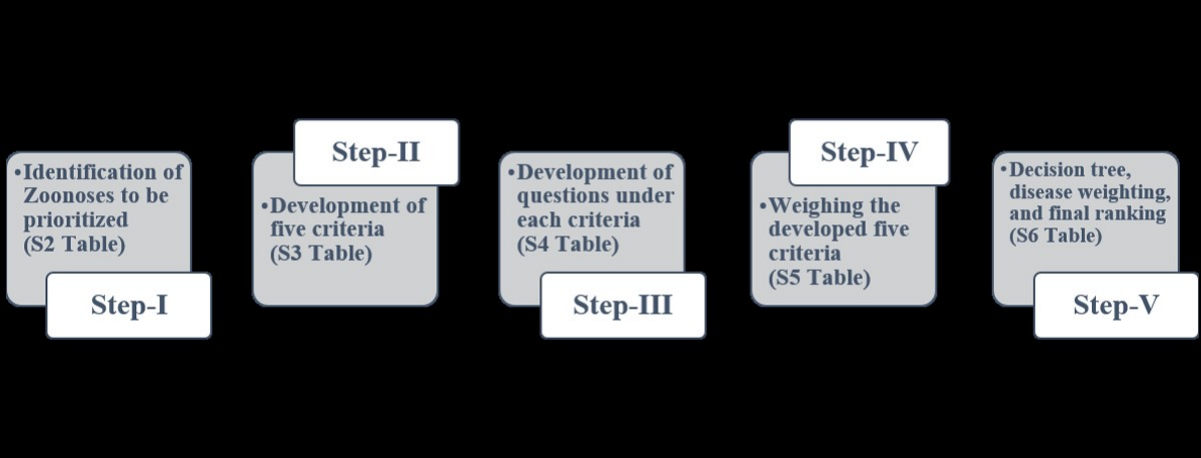
Schematic presentation of the steps involved in the prioritization process in Ahmedabad, Western city of India during September 2018.

With respect to the feasibility in the local context, we adopted these steps for prioritization as shown in Fig 1.

### Step I (Identification of zoonoses to be prioritized)

Prior to the workshop, a list of 33 zoonotic diseases relevant to Ahmedabad was developed. This list was developed based on informal discussions with five imperative stakeholders and literature search. The literature search included website searches of human and animal health organizations involved in zoonotic disease prevention and control, including national organizations, inter-governmental organizations, provincial organizations and academic institutions; reference textbooks and PubMed cataloged peer-reviewed publications without any time frame. This search aimed to compile all possible zoonotic diseases that was performed one month prior to the participatory workshop relevant to the local level.

Key search terms used included the disease criterion, the scientific and/or common name of diseases, and a combination of the two (e.g. case-fatality rate and/or brucellosis). The literature search was not a comprehensive literature review, but a focused search to compile the diseases. Further, at the beginning of the workshop, stakeholders were requested to enrich the list, if they felt any disease of local relevance was missing (S2 Table).

### Step II (Development of five criteria)

This step involved the development of five criteria, which were used to rank the importance of each zoonosis. These criteria were agreed upon during the workshop through a moderated discussion. Initially, the criteria used previously at different sites ([20], [21], [22], [23], [24], [25]) were reviewed and summarized. A list of eight criteria was provided to each stakeholder and each was requested to indicate the most relevant, while also giving them the chance to extend the list. The rank of each criterion, provided by the stakeholders, was averaged. The five criteria with the highest average rank were used for prioritization (S3 Table).

### Step III (Development of questions for each criterion)

This step involved a group discussion among the participants to develop questions to operationalize the criteria developed in step II. During the group discussion, five questions were developed, which were either binomial or multinomial. The answers of binomial questions were either yes or no. The multinomial questions had the following options: None (does not exist in any of the systems); Either (exists in any one of the systems); Both (exists in both the systems) (S4 Table).

The different answer options were assigned scores by the stakeholders. The score for each answer was guided by a group discussion. For each binomial question, ‘no’ and ‘yes’ were scored as 0 and 1 respectively. In multinomial questions, ‘none’, ‘either, or ‘both’ were scored as 0, 1 and 2 respectively. To avoid complications, we used neither ordinal scale questions nor specified cut-off values unlike other prioritization workshops [34,35]. In case of discrepancy, the question was further discussed until consensus was reached.

### Step IV (weighing the criteria)

Using the OHZDP tool Microsoft Excel spreadsheet [14], a semi-quantitative analytic hierarchy process was applied to assign the most important criteria with the highest weight, and the least important criteria with the lowest weight [14,19,36]. For this purpose, we divided the participants into groups of three to four, each group having a representative from each sector, thus forming six well-balanced groups. Although the process of OHZDP tool states that each member individually needs to rank the criteria, here a group exercise was applied, as we intend to have a common consensus across the sectors. Subsequently, each group ranked the five criteria according to their importance on a scale of 1 to 9, as previously done by another research group [36]. The group results were combined to produce the overall rank and weight of each criterion through an approximation method [37]. Regardless of how many factors were involved in making the decision, the approximation method only compares pairwise priorities for the criterion to calculate the overall weights. By doing this, we assessed the consistency of responses after combining them, ensuring adherence to both completeness and transitivity among the group choices for each criterion as per the Analytical Hierarchical Process (AHP). [14,17]. A consistency ratio of 0.01 or less was considered satisfactory (S5 Table).

### Step V (Decision-tree, disease weighting, and final ranking)

In accordance with the decision tree approach of the OHZDP tool, each group scored each of the 14 zoonotic diseases for each criterion. For example, the criterion ‘severity of disease in humans’ for rabies had the question “Does the disease cause morbidity and/or mortality among humans?”; if all agreed to option ‘No’ then that question received ‘0’, if all agreed to option ‘Yes’ then the question received ‘1’. The final score of the criterion was the sum of scores from all questions for the criterion. Two different total scores were calculated for each criterion i.e. weighted and unweighted. The calculation of the unweighted score simply uses the average of responses, while in the weighted final score the criteria weights assigned in step IV were applied (S6 Table).

For example: for the criterion ‘severity of disease in humans’ for rabies, all agreed on ‘Yes’ for the first question, so the unweighted score of the criterion was 1, whereas the weighted score of the criterion was 5 (as the criterion ‘severity of disease on humans’ received the rank 5). The final weighted score of the disease was then calculated by summing the product of the weight of each criterion with its unweighted score, obtained by averaging the scores of all the questions. For example, the final score for rabies was 15. Both the weighted and unweighted final scores of each disease were then normalized to the highest scoring disease, which consequently received a score of ‘1’. All workshop participants reviewed the disease-ranking results, which facilitated further discussion. The stakeholders then, through a facilitated discussion, collectively finalized the priority ranking of zoonotic diseases for Ahmedabad. During the facilitated discussion, if 2/3 of stakeholders agreed to a consensus, it was accepted.

### Sensitivity analysis

In the sensitivity analysis, the robustness of the prioritization outcome was assessed. In this step, three types of sensitivity analysis were conducted.

1. We assigned the five selected criteria equal weights and assessed how normalized disease scores compared to weighted disease scores.
2. A reverse weighting of the five criteria were done and normalized scores were compared.
3. We systematically removed each of the five developed criteria and assessed normalized disease scores with the four remaining criteria.

Pearson’s product-correlation coefficient was used to assess the relationships between these three normalized disease scores, with a coefficient p-value <0.01 considered significant. The analysis was conducted in R version 3.4.1 [38].

### Ethics approval

Ethics approval has been obtained from the Research Ethics Committee, Center for Development Research (ZEF), University of Bonn, Germany, and the Institutional Ethics Committee of the Indian Institute of Public Health Gandhinagar, India.

## Results

Out of 38 zoonoses included in the present study, stakeholders individually voted for diseases that should be used in the next steps of the workshop. At the end of step I, the number of diseases were reduced to 14 (Table 1), by averaging the votes of the stakeholders. The following five criteria were developed and presented from high to low importance:

1. Severity of disease in humans
2. Potential for epidemic/pandemic in humans and/or animals
3. Existence of prevention and control strategies in the human and/or animal health system
4. The burden of disease in animals
5. Existence of inter-sectoral collaboration for the disease

The results of the group exercise for weighting the criteria are shown in Table 2.

**Table 1:** Normalized weighted score of prioritized zoonotic diseases of Ahmedabad, Western city of India during participatory workshop, September 2018.

Zoonotic disease	Normalized Weighted Score
Rabies	1.000
Brucellosis	1.000
Crimean-Congo Hemorrhagic Fever (CCHF)	0.867
Avian Influenza (H5N1)	0.856
Influenza A (H1N1)	0.822
Tuberculosis	0.800
Salmonellosis	0.789
Japanese Encephalitis	0.767
Leptospirosis	0.722
Plague	0.722
Chikungunya	0.656
Dengue	0.633
Anthrax	0.400
Cholera	0.356

**Table 2:** Group ranking of criteria using the analytic hierarchy process from the prioritization workshop of Ahmedabad, Western city of India during September 2018.

Criteria	Group-1^#^	Group-2^#^	Group-3^#^	Group-4^#^	Group-5^#^	Group-6^#^	Overall Ranking^#^
Severity of Disease in Humans	0.03 (5)	0.42 (1)	0.56 (1)	0.29 (2)	0.53 (1)	0.52 (1)	0.223 (1)
Potential for Epidemic and/or Pandemic	0.13 (3)	0.04 (5)	0.10 (3)	0.48 (1)	0.30 (2)	0.26 (2)	0.207 (2)
Prevention and Control strategy	0.58 (1)	0.06 (4)	0.26 (2)	0.14 (3)	0.08 (3)	0.13 (3)	0.206 (3)
Burden of animal disease	0.17 (2)	0.25 (2)	0.06 (4)	0.04 (5)	0.06 (4)	0.06 (4)	0.184 (4)
Existing inter-sectoral collaboration	0.09 (4)	0.22 (3)	0.03 (5)	0.06 (4)	0.04 (5)	0.04 (5)	0.178 (5)
Consistency Ratio*	0.09	0.05	0.2	0.07	0.1	0.09	NA

(*) A consistency ratio of <0.1 is acceptable (Group 3 & 5 were excluded from the approximation for the final weights)

(#) Score gained during the Analytical Hierarchy process (Individual group rank)

With the help of a decision-tree analysis, the weight of each criterion was applied and a final weighted score was obtained to rank the diseases, which is shown in Table 1. Based on discussion, the stakeholders reached a consensus that the top two diseases remain unchanged, however, there was a change in the priority of other prioritized diseases (Table 3). This adjustment was done in view of the emerging cases in the city as well considering the outbreak history.

**Table 3:** Final prioritized disease rankings one health zoonotic disease prioritization workshop from the Ahmedabad, Western city of India during September 2018.

Disease	Final Ranking
Rabies	1
Brucellosis	2
Avian Influenza (H5N1)	3
Influenza A (H1N1)	4
Crimean-Congo Hemorrhagic Fever	5

The city of Ahmedabad experienced an outbreak of avian influenza in 2017 [39] and is currently documenting a series of new cases of influenza A [40]. Therefore, the Crimean-Congo hemorrhagic fever was moved to the fifth priority, while H5N1 and H1N1 were ranked as third and fourth prioritized zoonotic diseases of the city respectively.

**Fig 2:**
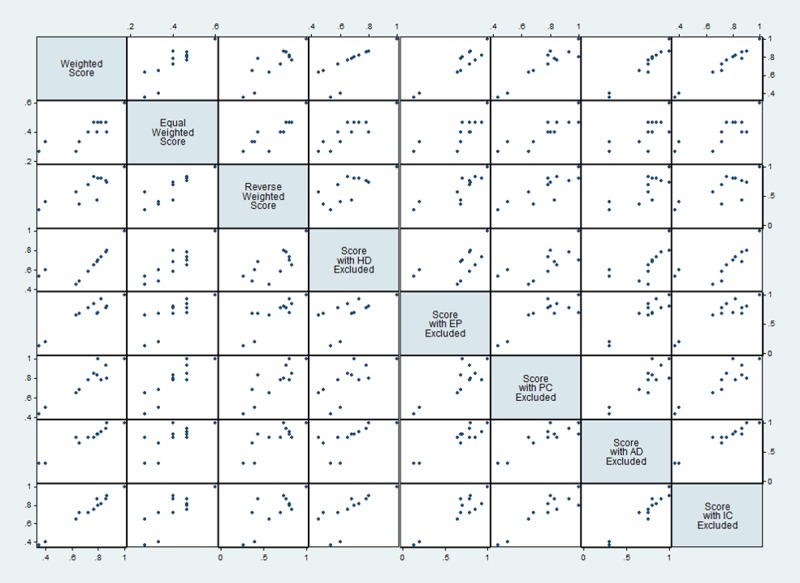
Comparison of normalized disease prioritization scores obtained from weighted criteria and with equal criteria weights, with reverse criteria weights and excluding each of the five criteria in the prioritization process in Ahmedabad, Western city of India during September 2018. (HD) Human disease, (EP) Epidemic potential, (PC) Prevention control, (AD) Animal disease, (IC) Intersectoral collaboration.

To assess the reliability of the finalized list a sensitivity analysis was conducted. Fig 2 indicates the sensitivity analysis with different strategies. The sensitivity analysis showed a strong positive correlation between scores produced by the OHZDP tool and normalized disease scores using equal weighted (r = 0.96, p <0.01) or reverse weighted criteria (r = 0.86, p <0.01). There was also a strong positive correlation when excluding each criterion, then comparing disease scores to those produced by the OHZDP tool (r = 0.89–0.99, p <0.01).

## Discussion

Participatory workshops for the prioritization of zoonotic diseases have been conducted in multiple countries, generating a unique list of priority zoonosis for each country. However, this is the first time such a workshop was conducted at a city level in India. The final list of priority zoonotic diseases in Ahmedabad was rabies, brucellosis, influenza (H5N1 & H1N1) and Crimean-Congo hemorrhagic fever. The prioritizations conducted at national level with the OHZDP tool had different objectives as per the need of the site. For example, Kenya conducted prioritization of zoonoses to provide guidelines for resource allocation to enhance surveillance, prevention, and control. Tanzania conducted zoonotic disease prioritization to understand which emerging zoonotic diseases should be jointly addressed through inter-ministerial collaboration. Comparing our findings to other sites such as Ethiopia, Tanzania and Congo; the top criterion was ‘severity of disease in humans’ in all these prioritization workshops, which indicates the strength and robustness of the process of OHZDP tool. The process of OHZDP tool helped to allocate resources, budgeting, and provide policy guidance. Further, to our knowledge this is the first study, which adopted the process of zoonotic disease prioritization through OHZDP tool at the local level.

In India, there are some efforts at the national level to prioritize zoonotic diseases in order to prioritize research needs for the control of zoonoses, such as the Roadmap to Combat Zoonoses in India (RCZI) [26] and the simple ranking of disease by Kurian *et al*. [27]. RCZI adopted the priority setting methods developed by the Child Health and Nutrition Research Initiative [26], whereas Kurian *et al*. adopted a composite index method based on the trends and distribution of each disease and their adverse effects on human health, economy, trade and industry [27]. The objective of these two prioritizations differed. For example, RCZI prioritized the zoonoses that should be given priority with respect to research in next decade, while Kurian *et al*. prioritized the zoonoses based on the burden of disease in India. There are various limitations to the methodologies used in these prioritizations. For example, the RCZI method involved an assumption that the Child Health and Nutrition Research Initiative's (CHNRI) five recommended scoring criteria are also applicable to the Indian zoonoses context. Moreover, they represent the key metrics that stakeholders would use to prioritize research options rather than taking a disease burden point of view.

The challenges posed by children's health issues, for which the CHNHRI was originally developed, may be substantially different from those posed by zoonoses prioritization used by RCZI group. Similarly, the composite index method used by Kurian *et al*., usually requires exact data to measure the disease burden. Considering the zoonoses database and surveillance system in India, there is a lack of zoonotic data at the national and local level, thus the approach followed in this current study is better suited to setting with low data availability. Nonetheless, the past Indian zoonotic disease prioritizations done by the RCZI and Kurian *et al*. were compared with the prioritization of the current study conducted at the local level and is summarized in Table 4. Like other global sites, prioritizations in India also ranked rabies as the top priority irrespective of the goal and method of the workshops. In addition, brucellosis ranks high in all three models. Interestingly leptospirosis received a high rank at the national level but was only in the last place at the local level; this highlights that diseases are context-specific and need to be assessed locally in order to develop target-oriented interventions. An important observation from this exercise is that local priorities may be different from national aggregated priorities, which emphasizes the need for this type of prioritization at each local setting.

**Table 4:** Summary of prioritized zoonotic diseases in India with respect to time, region and aim of prioritization.

Level	National (India)	National (India)	Local (Ahmedabad)
**Author**	Sekar et al., March 2009	Kurian et al., September 2013	Current Study, September 2018
**Goal**	To prioritize research options needed to control zoonoses.	To identify and rank the most important zoonotic diseases in India.	To determine which zoonoses should receive high concern for collaboration between the stakeholders in a smart city of India, Ahmedabad.
**Method**	Child Health and Nutrition Research Initiative’s priority setting method.	Composite index method based on the trends of disease, adverse effects on human health, economy, trade and industry.	Centers for Disease Control and Prevention’s One Health Zoonotic Disease Prioritization tool.
**Prioritized diseases in descending order**	Rabies, Leptospirosis, Brucellosis, Anthrax, Tuberculosis, Pandemic Flu, Helminths, Arbovirus, Food borne	Rabies, Avian Influenza (H5N1), Anthrax, Brucellosis, Leptospirosis, Tuberculosis, Japanese encephalitis, Porcine cysticercosis	Rabies, Brucellosis, Avian Influenza (H5N1), Influenza A (H1N1), Crimean-Congo Hemorrhagic Fever, Tuberculosis, Salmonellosis, Japanese encephalitis, Leptospirosis

The OHZDP tool that the current study adopted as the prime tool for prioritization has certain limitations that became evident during the process. The selection of prioritization criteria are specific to the workshop participants and the weighting and scoring of these is highly affected by the participants and their background. It is important to note that when diverse stakeholders such as health officials and administrators come together, some bias is introduced, including group thinking and politics; however, a strong moderator can overcome these by focusing on the key objective of the workshop and creating a single platform. The questions chosen for the evaluations of the criteria are also highly dependent on the workshop participants and may not be applicable to the impact of all zoonoses. From this study, we learnt that although avoiding non-ordinal questions makes the OHZDP process quicker and easily palatable to a diverse range of stakeholders, it leads to less robust results, as the severity of human health is only scored yes/no for example, giving no room to highlight the differing burden of different diseases. Another modification was the use group ranking in the AHP process (Step-IV) rather than individual ranking, because we intended to develop a common consensus across the sectors. Therefore, each group consisted of stakeholders from the different sectors. Such debate between the sectors has to be considered at the time of planning of a similar kind of participatory workshop for disease prioritization, if the group exercise is considered as part of the AHP at the local level. When the same tool is applied at national level then there must be more options to include diverse stakeholders; however, at the local level, the numbers of stakeholders are much limited and it becomes a challenge when a particular stakeholder is unable to make it on the day of workshop. Therefore, considering the flexible nature of OHZDP, we recommend these changes while adopting at the local level.

## Conclusion

Prioritization of zoonotic diseases on the local level is essential for development of One Health strategies. In addition to its established usefulness at national level, the OHZDP tool of the CDC can also assist local policy makers or program managers to make such prioritization to facilitate better planning and collaboration. The prioritization of diseases can vary according to the aim of the participatory workshop, as the aim affects the criteria selection and scoring of diseases. It is therefore very important to highlight the main goal of the workshop to the participants in order to achieve the desired outcomes. The selection of the workshop participants is also highly important and attention should be paid to engaging a wide range of stakeholders and balancing stakeholders from different sectors and with different expertise. This type of participatory workshop for disease prioritization is highly recommended and can be replicated in other cities in India or in other lower-middle income countries. Among others, this study concludes that OHZDP tool can be adopted to local level, provided the stakeholders are selected carefully as per the objective of collaborative disease prioritization.

## Supporting information

S1 Table List of anonymized stakeholders who have participated in the zoonotic disease prioritization in Ahmedabad, Western city of India during participatory workshop, September 2018. (*) Public, (#) Private/ Non-Governmental Organization
(AMC) Ahmedabad Municipal Corporation, (GVC) Gujarat Veterinary Council, (CNCD) Cattle Nuisance Control Department, (DP) District Panchayat Office, (ADIO) Animal Disease Investigation Office
(DOCX)

S2 Table List of zoonotic diseases for the prioritization in Ahmedabad, Western city of India during participatory workshop, September 2018. (Note) An initial list of diseases were compiled from literature review and informal discussion with experts. The open list of the diseases were provided to the participants and asked to select the relevant diseases in context to Ahmedabad, which need to be prioritized.
(S) Stakeholder
(DOCX)

S3 Table Deciding the criteria for the prioritization in Ahmedabad, Western city of India during the participatory workshop, September 2018. (S) Stakeholder
(DOCX)

S4 Table Questionnaires developed under each criterion for the prioritization of zoonotic diseases in Ahmedabad, Western city of India during the participatory workshop, September 2018. (OIE) World Organization for Animal Health
(DOCX)

S5 Table Group ranking of criteria for prioritizing zoonotic diseases using the Analytic Hierarchy Process in Ahmedabad, Western city of India during the participatory workshop, September 2018. (HD) Severity of disease in humans, (PC) Prevention and Control strategy, (EP) Potential for Epidemic and/or Pandemic, (AD) Burden of animal disease, (IC) Existing inter-sectoral collaboration
(DOCX)

S6 Table Weighing of prioritized zoonotic diseases using decision tree analysis fin Ahmedabad, Western city of India during the participatory workshop, September 2018. (HD) Severity of disease in humans, (PC) Prevention and Control strategy, (EP) Potential for Epidemic and/or Pandemic, (AD) Burden of animal disease, (IC) Existing inter-sectoral collaboration
(DOCX)
