# Supplementary material for: Multi-sectoral prioritization of zoonotic diseases: One health perspective from Ahmedabad, India
Source: PLoS One. 2019 Jul 30;14(7):e0220152. doi: 10.1371/journal.pone.0220152 (PMC6667134; doi:10.1371/journal.pone.0220152)
Supplement: S1 Table — (*) Public, (#) Private/ Non-Governmental Organization (AMC) Ahmedabad Municipal Corporation, (GVC) Gujarat Veterinary Council, (CNCD) Cattle Nuisance Control Department, (DP) District Panchayat Office, (ADIO) Animal Disease Investigation Office (DOCX) [file pone.0220152.s001.docx]

**S1 Table. List of anonymized stakeholders who have participated in the zoonotic disease prioritization in Ahmedabad, Western city of India during participatory workshop, September 2018**

| **Designation** | **Department** | **Sector** |
| --- | --- | --- |
| Deputy Health Officer, Epidemic | Health, AMC | Human Health^*^ |
| Registrar | GVC | Animal Health^*^ |
| Superintendent | CNCD, AMC | Animal Health ^*^ |
| Assistant Health Officer, Urban Health | Health, AMC | Human Health^*^ |
| Deputy Director | Animal Husbandry, DPO | Animal Health ^*^ |
| Deputy Director | Animal Husbandry, ADIO | Animal Health ^*^ |
| Microbiologist | Academia | Human Health^*^ |
| Veterinarian | Private Practitioner | Animal Health ^#^ |
| Veterinarian Officer | NGO/Trust Hospital | Animal Health ^#^ |
| Manager | Animal Rights Activists | Animal Health ^#^ |
| Malaria Officer | Health, DPO | Human Health^*^ |
| Entomologist | Health, AMC | Human Health^*^ |
| Director | Zoo, Ahmedabad | Animal Health ^*^ |
| Surveillance Officer | Health, Gujarat State | Human Health^*^ |
| Associate Professor, Professor | Academic Institutes | Human Health^*#^ |

*(*) Public, (#) Private/ Non-Governmental Organization*

*(AMC) Ahmedabad Municipal Corporation, (GVC) Gujarat Veterinary Council, (CNCD)Cattle Nuisance Control Department, (DP) District Panchayat Office, (ADIO) Animal Disease Investigation Office*
