# Supplementary material for: Multi-sectoral prioritization of zoonotic diseases: One health perspective from Ahmedabad, India
Source: PLoS One. 2019 Jul 30;14(7):e0220152. doi: 10.1371/journal.pone.0220152 (PMC6667134; doi:10.1371/journal.pone.0220152)
Supplement: S4 Table — (OIE) World Organization for Animal Health (DOCX) [file pone.0220152.s004.docx]

**S4 Table. Questionnaires developed under each criterion for the prioritization of zoonotic diseases in Ahmedabad, Western city of India during the participatory workshop, September 2018**

| **Criteria** | **Question** |
| --- | --- |
| Severity of Disease in Humans (HD) | Does the disease cause morbidity and/or mortality among humans? (0. No, 1.Yes) |
| Prevention and Control strategy (PC) | Is there an effective control strategy in both humans and animals in Ahmedabad? (0. Neither, 1. Either, 2. Both) |
| Potential for Epidemic and/or Pandemic (EP) | Has the disease caused an epidemic in humans or animals in the last 10 years in Ahmedabad? (0. Neither, 1. Either, 2. Both) |
| Burden of animal disease (AD) | Is the disease considered as burden for animals? (0. Disease not present, OIE not reportable, 1. Disease not present but OIE reportable, 2. Disease present, OIE not reportable, 3. Disease present & OIE reportable) |
| Existing inter-sectoral collaboration (IC) | Is there any inter-sectoral collaboration existing among human and animal health system in Ahmedabad? (0.No, 1.Yes) |

*(OIE) World Organization for Animal Health*
