# Supplementary material for: Multi-sectoral prioritization of zoonotic diseases: One health perspective from Ahmedabad, India
Source: PLoS One. 2019 Jul 30;14(7):e0220152. doi: 10.1371/journal.pone.0220152 (PMC6667134; doi:10.1371/journal.pone.0220152)
Supplement: S5 Table — (HD) Severity of disease in humans, (PC) Prevention and Control strategy, (EP) Potential for Epidemic and/or Pandemic, (AD) Burden of animal disease, (IC) Existing inter-sectoral collaboration (DOCX) [file pone.0220152.s005.docx]

**S5 Table. Group ranking of criteria for prioritizing zoonotic diseases using the Analytic Hierarchy Process in Ahmedabad, Western city of India during the participatory workshop, September 2018**

| **Group** |  | **HD** | **PC** | **EP** | **AD** | **IC** |
| --- | --- | --- | --- | --- | --- | --- |
| **Group 1** | **HD** | 1.00 | 0.11 | 0.17 | 0.14 | 0.20 |
|  | **PC** | 9.00 | 1.00 | 8.00 | 5.00 | 7.00 |
|  | **EP** | 6.00 | 0.13 | 1.00 | 1.00 | 2.00 |
|  | **AD** | 7.00 | 0.20 | 1.00 | 1.00 | 3.00 |
|  | **IC** | 5.00 | 0.14 | 0.50 | 0.33 | 1.00 |
| **Group 2** | **HD** | 1.00 | 9.00 | 8.00 | 2.00 | 2.00 |
|  | **PC** | 0.11 | 1.00 | 2.00 | 0.20 | 0.25 |
|  | **EP** | 0.13 | 0.50 | 1.00 | 0.25 | 0.11 |
|  | **AD** | 0.50 | 5.00 | 4.00 | 1.00 | 2.00 |
|  | **IC** | 0.50 | 4.00 | 9.00 | 0.50 | 1.00 |
| **Group 3** | **HD** | 1.00 | 9.00 | 7.00 | 8.00 | 9.00 |
|  | **PC** | 0.11 | 1.00 | 5.00 | 9.00 | 8.00 |
|  | **EP** | 0.14 | 0.20 | 1.00 | 2.00 | 5.00 |
|  | **AD** | 0.13 | 0.11 | 0.50 | 1.00 | 3.00 |
|  | **IC** | 0.11 | 0.13 | 0.20 | 0.33 | 1.00 |
| **Group 4** | **HD** | 1.00 | 4.00 | 0.33 | 8.00 | 6.00 |
|  | **PC** | 0.25 | 1.00 | 0.20 | 5.00 | 4.00 |
|  | **EP** | 3.00 | 5.00 | 1.00 | 8.00 | 7.00 |
|  | **AD** | 0.13 | 0.20 | 0.13 | 1.00 | 0.50 |
|  | **IC** | 0.17 | 0.25 | 0.14 | 2.00 | 1.00 |
| **Group 5** | **HD** | 1.00 | 5.00 | 5.00 | 9.00 | 9.00 |
|  | **PC** | 0.20 | 1.00 | 0.13 | 2.00 | 2.00 |
|  | **EP** | 0.20 | 8.00 | 1.00 | 5.00 | 9.00 |
|  | **AD** | 0.11 | 0.50 | 0.20 | 1.00 | 2.00 |
|  | **IC** | 0.11 | 0.50 | 0.11 | 0.50 | 1.00 |
| **Group 6** | **HD** | 1.00 | 5.00 | 4.00 | 8.00 | 9.00 |
|  | **PC** | 0.20 | 1.00 | 0.20 | 3.00 | 5.00 |
|  | **EP** | 0.25 | 5.00 | 1.00 | 4.00 | 7.00 |
|  | **AD** | 0.13 | 0.33 | 0.25 | 1.00 | 2.00 |
|  | **IC** | 0.11 | 0.20 | 0.14 | 0.50 | 1.00 |

*(HD) Severity of disease in humans, (PC) Prevention and Control strategy, (EP) Potential for Epidemic and/or Pandemic, (AD) Burden of animal disease, (IC) Existing inter-sectoral collaboration*
