# Supplementary material for: Multi-sectoral prioritization of zoonotic diseases: One health perspective from Ahmedabad, India
Source: PLoS One. 2019 Jul 30;14(7):e0220152. doi: 10.1371/journal.pone.0220152 (PMC6667134; doi:10.1371/journal.pone.0220152)
Supplement: S6 Table — (HD) Severity of disease in humans, (PC) Prevention and Control strategy, (EP) Potential for Epidemic and/or Pandemic, (AD) Burden of animal disease, (IC) Existing inter-sectoral collaboration (DOCX) [file pone.0220152.s006.docx]

**S6 Table. Weighing of prioritized zoonotic diseases using Decision Tree analysis fin Ahmedabad, Western city of India during the participatory workshop, September 2018**

| **Prioritized Zoonotic diseases** | **Initial Scores** | | | | | **Weighted scores (by criterion weight)** | | | | | **Weighted Score** | **Normalized Weighted Score** |
| --- | --- | --- | --- | --- | --- | --- | --- | --- | --- | --- | --- | --- |
|  | **HD** | **EP** | **PC** | **AD** | **IC** | **HD** | **EP** | **PC** | **AD** | **IC** |  |  |
| Dengue | 1 | 1 | 1 | 0 | 1 | 5 | 2.00 | 1.50 | 0.00 | 1.00 | 9.50 | 0.633 |
| Rabies | 1 | 2 | 2 | 3 | 1 | 5 | 4.00 | 3.00 | 2.00 | 1.00 | 15.00 | 1.000 |
| Swine Flu (H1N1) | 1 | 1 | 2 | 2 | 1 | 5 | 2.00 | 3.00 | 1.33 | 1.00 | 12.33 | 0.822 |
| Tuberculosis | 1 | 2 | 0 | 3 | 1 | 5 | 4.00 | 0.00 | 2.00 | 1.00 | 12.00 | 0.800 |
| Chikungunya | 1 | 1 | 1 | 2 | 0 | 5 | 2.00 | 1.50 | 1.33 | 0.00 | 9.83 | 0.656 |
| Avian Influenza (H5N1) | 1 | 2 | 1 | 2 | 1 | 5 | 4.00 | 1.50 | 1.33 | 1.00 | 12.83 | 0.856 |
| Salmonellosis | 1 | 2 | 1 | 2 | 0 | 5 | 4.00 | 1.50 | 1.33 | 0.00 | 11.83 | 0.789 |
| Brucellosis | 1 | 2 | 2 | 3 | 1 | 5 | 4.00 | 3.00 | 2.00 | 1.00 | 15.00 | 1.000 |
| [Crimean-Congo Hemorrhagic Fever (CCHF)](https://www.cdc.gov/vhf/crimean-congo/index.html) | 1 | 2 | 2 | 0 | 1 | 5 | 4.00 | 3.00 | 0.00 | 1.00 | 13.00 | 0.867 |
| Leptospirosis | 1 | 1 | 1 | 2 | 1 | 5 | 2.00 | 1.50 | 1.33 | 1.00 | 10.83 | 0.722 |
| Vibrio Cholera | 0 | 2 | 0 | 2 | 0 | 0 | 4.00 | 0.00 | 1.33 | 0.00 | 5.33 | 0.356 |
| Japanese Encephalitis | 1 | 1 | 1 | 3 | 1 | 5 | 2.00 | 1.50 | 2.00 | 1.00 | 11.50 | 0.767 |
| Plague | 1 | 1 | 1 | 2 | 1 | 5 | 2.00 | 1.50 | 1.33 | 1.00 | 10.83 | 0.722 |
| Anthrax | 0 | 2 | 0 | 3 | 0 | 0 | 4.00 | 0.00 | 2.00 | 0.00 | 6.00 | 0.400 |

*(HD) Severity of disease in humans, (PC) Prevention and Control strategy, (EP) Potential for Epidemic and/or Pandemic, (AD) Burden of animal disease, (IC) Existing inter-sectoral collaboration*
